# Atypical Pulmonary Tuberculosis as the First Manifestation of Advanced HIV Disease—Diagnostic Difficulties

**DOI:** 10.3390/diagnostics12081886

**Published:** 2022-08-04

**Authors:** Aneta Kacprzak, Karina Oniszh, Regina Podlasin, Maria Marczak, Iwona Cielniak, Ewa Augustynowicz-Kopeć, Witold Tomkowski, Monika Szturmowicz

**Affiliations:** 1 1st Department of Lung Diseases, National Tuberculosis and Lung Diseases Institute, Plocka 26, 01-138 Warsaw, Poland; 2 Radiology Department, National Tuberculosis and Lung Diseases Institute, 01-138 Warsaw, Poland; 3 4th Department of Infectious Diseases, Hospital for Infectious Diseases in Warsaw, 01-201 Warsaw, Poland; 4 1st Department of Infectious Diseases, Hospital for Infectious Diseases in Warsaw, 01-201 Warsaw, Poland; 5 Department of Microbiology, National Tuberculosis and Lung Diseases Institute, 01-138 Warsaw, Poland

**Keywords:** tuberculosis, human immunodeficiency virus, acquired immunodeficiency syndrome, chest imaging

## Abstract

Tuberculosis (TB) is the leading cause of morbidity, hospitalisations, and mortality in people living with HIV (PLWH). The lower CD4+ T-lymphocyte count in the course of HIV infection, the higher risk of active TB, and the higher odds for atypical clinical and radiologic TB presentation. These HIV-related alterations in TB presentation may cause diagnostic problems in patients not knowing they are infected with HIV. We report on a patient without any background medical conditions, who was referred to a hospital with a 4-month history of chest and feet pains, mild dry cough, fatigue, reduced appetite, and decreasing body weight. Chest X-ray revealed mediastinal lymphadenopathy, bilateral reticulonodular parenchymal opacities, and pleural effusion. A preliminary diagnosis of lymphoma, possibly with a superimposed infection was established. Further differential diagnostic process revealed pulmonary TB in the course of advanced HIV-1 disease, with a CD4+ T-lymphocyte count of 107 cells/mm^3^. The patient completed anti-tuberculous therapy and successfully continues on antiretroviral treatment. This case underlines the importance of screening for HIV in patients with newly diagnosed TB.

**Figure 1:**
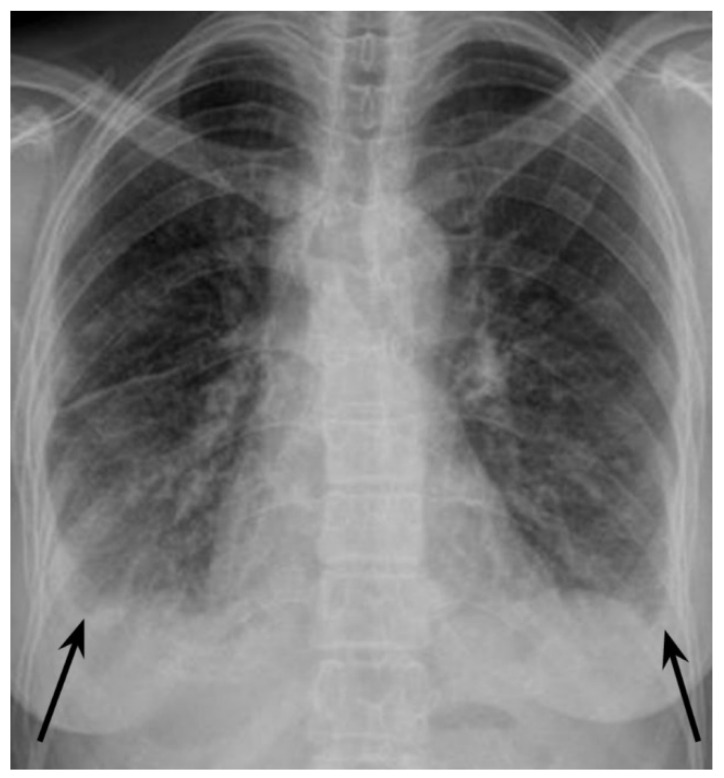
Infection with human immunodeficiency virus (HIV) is the strongest known risk factor for active tuberculosis (TB), and the risk of developing active TB in people living with HIV (PLWH) is 15–22 times higher than in people without HIV [1]. Active TB may develop at any stage of HIV infection, but the risk correlates negatively with CD4^+^ cells count. TB is the leading cause of morbidity, hospitalisations, and mortality in PLWH [1]. There were 214,000 deaths due to TB among HIV-positive people in 2020 worldwide, which accounted for 31.5% of all HIV-related deaths [1,2]. Therefore, it is recommended to screen for TB in HIV-positive patients, and for HIV infection in newly diagnosed TB patients [3,4,5]. Around 16% of all PLWH do not know that they are infected with HIV [1], and about 25% of incident HIV patients present to care with advanced disease [3]. Immunosuppression caused by HIV infection affects clinical and radiologic presentation of TB. Atypical TB presentation is often observed in the late stages of HIV infection [6,7,8,9]. Such atypical TB presentation in a person with HIV infection not yet diagnosed, may be challenging, as described below. A 42-year-old woman of Indian origin was referred to a respiratory medicine department after her chest X-ray (Figure 1) revealed nodular opacifications in the lungs and bilateral pleural effusion (arrows). The patient had a 4-month history of unspecific chest and feet pains, mild dry cough, fatigue, reduced appetite, and body weight loss of 6 kg. She denied dyspnoea, sputum expectoration, haemoptysis, night sweats, or fever. On admission to the hospital, she was in good condition, her vital signs were normal, BMI was 19.2. There was no palpable peripheral lymphadenopathy or oedema; the vesicular breathing sound was reduced bibasiliary on chest auscultation. Blood tests showed elevated CRP—109.4 (N:<5) mg/L and ERS—120 (N: < 12), normal procalcitonin, normal leukocyte and neutrophil counts, decreased lymphocyte count—0.84 × 10^3^ (N:1.18 × 10^3^–3.74 × 10^3^) cells/mm^3^.

**Figure 2:**
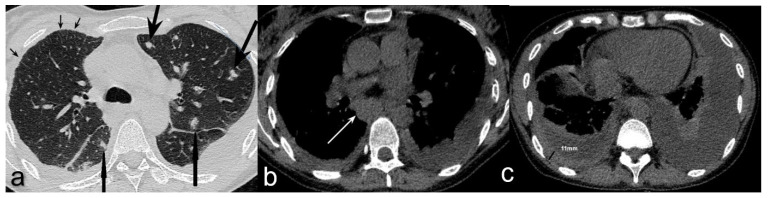
Axial non-contrast chest CT showed (**a**) multiple bilateral lung nodules (big arrows) and foci of thickened interlobular septa (small arrows); (**b**) enlarged subcarinal lymph nodes (white arrow); (**c**) right-sided pleural effusion with pleural thickening (measured) and left-sided pleural effusion partially compressing underlying lung parenchyma. Enlarged left supraclavicular and hepatic lymph nodes were found in the ultrasound examination. The overall clinical and radiologic appearance was suggestive of a lymphoma. On the day of admission, the patient developed fever of 40 °C. Sputum, blood, and urine cultures were taken—all turned out to be negative. Neither Legionella antigen in urine sample nor CMV antigen in blood were detected. An HIV test was requested as a part of the differential diagnosis of lymphopenia and lymphadenopathy. Meanwhile, an empirical antibiotic therapy consisting of ceftriaxone and levofloxacin was commenced; it was soon extended by the inclusion of clindamycin because the patient continued to be febrile. Bronchoscopy was performed and it showed a scar from previous lymph node ulceration into the left upper bronchus; aside from this the macroscopic appearance of the airways was irrelevant. The fluid from bronchoalveolar lavage (BALF) was collected and cultured. Direct microscopic examination was negative for acid-fast bacilli, but molecular testing with GeneXpert MTB/RIF assay detected the presence of *Mycobacterium tuberculosis complex* DNA; no rifampicin resistance gene was found. At the same time, a positive result for HIV-1 test (Bio-Rad Geenius ^TM^ HIV 1/2 Confirmatory Assay) was obtained. Anti-tuberculous treatment was started with isoniazid, rifampicin, ethambutol, and pyrazinamide. Soon, the patient became apyrexial and CRP started to decrease gradually. *Mycobacterium tuberculosis* (MTB) strain, susceptible to all primary anti-TB drugs, was cultured from BALF after two weeks. At the time of HIV infection diagnosis, the CD4^+^ T-lymphocyte count was 107 cells/mm^3^, and viral load 13,846 copies/mm^3^. Antiretroviral therapy (ART) with emtricitabine, tenofovir, and dolutegravir was commenced 11 days after anti-TB treatment was started. It resulted in a gradual improvement in CD4^+^ cell count and viral suppression; after four months CD4^+^ T-lymphocyte count reached level of >400 cells/mm^3^. Both anti-TB and antiviral therapies were well tolerated. The four-drug anti-TB treatment was continued for two months and was followed by seven months of isoniazid and rifampicin. Follow-up chest HRCT showed the regression of mediastinal lymphadenopathy and significant improvement of parenchymal and pleural abnormalities. The series of sputum cultures for acid-fast bacilli was negative. Three years after the diagnosis of pulmonary TB and acquired immunodeficiency syndrome (AIDS), the patient remains in good condition and continues ART successfully in an HIV/AIDS-dedicated out-patient clinic. The reported patient with newly diagnosed HIV infection and pulmonary TB had no background chronic conditions and considered herself healthy until the unspecific chest and feet pains, mild dry cough, fatigue, and reduced appetite occurred. The symptoms of pulmonary TB are unspecific, and the most common include cough with scanty sputum, haemoptysis, dyspnoea, chest pain, low-grade intermittent fever, sweating, fatigue, and weight loss [10]. Pulmonary TB in HIV-positive people presents with similar symptoms, but lower CD4^+^ cell counts are associated with more severe systemic symptoms [9,11]. Typical chest X-ray findings in pulmonary TB are: upper lung zones patchy consolidations, cavitations, features of bronchial dissemination [12]. Such radiologic features were not found in the presented patient. Chest CT scan showed multiple small nodules with perilymphatic distribution, interlobular septa thickening, enlarged mediastinal lymph nodes, and bilateral pleural effusion. As the HIV status of the patient was not known at that time, the radiologists suggested lung involvement in the course of a lymphoproliferative disease. That impression was further augmented by the presence of abdominal and supraclavicular lymphadenopathy. Nonspecific symptoms, high inflammatory indices, the presence of lymphopenia, lymphadenopathy, and pulmonary and pleural lesions required broad diagnostic work-up. Testing for HIV and MTB gave the conclusive outcome, and pulmonary TB in the course of HIV infection was diagnosed. Radiologic phenotype of TB associated with HIV is similar to that without HIV co-infection when CD4^+^ cell count is high, i.e., >350 cells/mm^3^. In cases with lower CD4^+^ cell counts, the presentation of pulmonary TB is shifted towards atypical patterns, such as parenchymal consolidations affecting the middle and lower lung zones, miliary infiltrates, chest lymphadenopathy, and pleural effusion, as in the presented patient [6,7,8,9]. Lung disease in the late period of HIV infection may be caused by opportunistic bacteria, tuberculous and non-tuberculous mycobacteria, viral and fungal pathogens, but also by the spectrum of neoplastic disorders, such as non-Hodgkin’s lymphoma or Kaposi’s sarcoma [13]. They may present with pleural effusion and pulmonary nodular infiltrates [13,14]. Thus, the differential considerations in the reported patient should also include these neoplastic diseases. The bronchoscopy revealed no suspicious endobronchial lesions, and microscopic examination of BALF was negative for atypical or neoplastic cells. Finally, therapies targeted at TB and HIV resulted in resolution of lymphadenopathy. An additional difficulty in diagnosing TB in PLWH is a higher rate of smear-negative disease. For this reason, molecular WHO-recommended rapid diagnostic tests, such as GeneXpert MTB/RIF assay, are recommended as an initial test rather than smear microscopy or culture [3]. Anti-tuberculous treatment regimen and its duration in drug-susceptible TB in PLWH are the same as in TB without HIV co-infection [5]. ART should be started as soon as possible (within two weeks of initiating TB treatment) regardless of CD4^+^ cell count, with the only exception being TB meningitis, where it should be delayed for four weeks if CD4^+^ < 50 (100) cells/mm^3^ [5]. The risk of death in people co-infected with HIV and TB is reportedly two to five times higher than in HIV-infected patients without TB with matched CD4^+^ cell counts, irrespective of ART, effective TB treatment, and good access to healthcare [15,16,17]. There is the evidence that TB directly contributes to mortality in HIV-infected patients, rather than simply presenting as a marker of advanced immunodeficiency [18]. Delayed TB diagnosis, i.e., after ≥1 month of symptoms duration, increases the risk of death [19], and the first 3 months after TB diagnosis seem crucial for survival in PLWH [15]. A TB recurrence rate is higher in PLWH than in people without HIV—4.5 vs. 1.9 per 100 person-years, respectively [20]. The presented patient remains well three years after diagnosis of TB and HIV co-infection, with viral suppression on ART and no signs of TB recurrence. Populations with high prevalence of HIV infection include men who have sex with men, intravenous drug users, people in prisons and other closed settings, sex workers, and transgender people [21]. In summary, co-infection with HIV may change the clinical phenotype of TB, leading to diagnostic problems and delayed treatment. The highest level of vigilance with regard to TB is recommended in PLWH. Moreover, each newly diagnosed TB patient should be tested for HIV.

## Data Availability

Not applicable.
